# Accurate drusen segmentation in optical coherence tomography via order-constrained regression of retinal layer heights

**DOI:** 10.1038/s41598-023-35230-4

**Published:** 2023-05-19

**Authors:** Olivier Morelle, Maximilian W. M. Wintergerst, Robert P. Finger, Thomas Schultz

**Affiliations:** 1 B-IT and Department of Computer Science, University of Bonn, 53115 Bonn, Germany; 2 Department of Ophthalmology, University Hospital Bonn, 53127 Bonn, Germany; 3 Lamarr Institute for Machine Learning and Artificial Intelligence, https://lamarr-institute.org

**Keywords:** Computer science, Macular degeneration, Biomarkers, Biomarkers, Computational science

## Abstract

Drusen are an important biomarker for age-related macular degeneration (AMD). Their accurate segmentation based on optical coherence tomography (OCT) is therefore relevant to the detection, staging, and treatment of disease. Since manual OCT segmentation is resource-consuming and has low reproducibility, automatic techniques are required. In this work, we introduce a novel deep learning based architecture that directly predicts the position of layers in OCT and guarantees their correct order, achieving state-of-the-art results for retinal layer segmentation. In particular, the average absolute distance between our model’s prediction and the ground truth layer segmentation in an AMD dataset is 0.63, 0.85, and 0.44 pixel for Bruch's membrane (BM), retinal pigment epithelium (RPE) and ellipsoid zone (EZ), respectively. Based on layer positions, we further quantify drusen load with excellent accuracy, achieving 0.994 and 0.988 Pearson correlation between drusen volumes estimated by our method and two human readers, and increasing the Dice score to 0.71 ± 0.16 (from 0.60 ± 0.23) and 0.62 ± 0.23 (from 0.53 ± 0.25), respectively, compared to a previous state-of-the-art method. Given its reproducible, accurate, and scalable results, our method can be used for the large-scale analysis of OCT data.

## Introduction

Age-related macular degeneration (AMD) is the main cause of severe vision loss and blindness in Europe and has a prevalence of 25% in those over 60. Due to the demographic shift, the number of affected patients is projected to increase by 15% by 2050^1^. In order to meet the corresponding increase in demand for retinal imaging and its analysis, automatic image analysis of retinal images may provide valuable support with diagnoses and monitoring of patients which is important for effective treatment and may also enable the reproducible evaluation of biomarkers in epidemiological studies. A hallmark biomarker of AMD is drusen, defined as deposits between the Retinal Pigment Epithelium (RPE) and the Bruch's Membrane (BM)^2^.

Optical Coherence Tomography (OCT) is a method to non-invasively capture three-dimensional imaging data of the retina using a sequence of equally spaced depth scans (B-scans). While such data enables the accurate quantification of retinal biomarkers such as drusen, the grading process is time-consuming and benefits from automated approaches which improve reproducibility, comparability across studies and reduce associated costs.Therefore, several conventional machine learning^3^ and deep learning-based OCT segmentation models exist. Most of them approach the problem as a semantic segmentation problem where segmentation masks of the same size as the input image are predicted. Existing models differ in architecture or loss functions and focus on the segmentation of different biomarkers like drusen^4–7^, hyperreflective foci (HRF)^8,9^, or fluid in or below the retina^10^. Treating the localization of retinal layers in OCT as a semantic segmentation problem is attractive due to the maturity and ease of use of corresponding neural network architectures. However, it does not easily support end-to-end learning, since our final goal is not pixel-wise labeling, but rather finding the correct height of each layer in each column (A-scan) of the overall slice image (B-scan). In most existing approaches, this requires an additional post-process. Moreover, this makes it difficult to integrate helpful anatomical prior knowledge, particularly concerning the order in which the different layers occur.

Before the wide adoption of deep learning, state-of-the-art approaches^11^ directly determined layer height and enforced the correct layer ordering. The direct prediction of height has also been attempted with convolutional neural networks by treating layer localization as a 2D-to-1D regression problem^12,13^ in which, more recently, layer order has again been considered^14^. It has also been tried to frame the segmentation as a sequence labeling problem on A-scans, an approach that lets the model learn a prior about the layer order^15^.

In this study, we introduce a novel CNN architecture that first segments areas above and below the respective layers under constraints that guarantee their correct ordering. It then passes the intermediate segmentation result through a so-called layer head that computes the layer heights from it. It can be used for end-to-end learning of retinal layers with a loss that directly penalizes deviations from their correct position. On a frequently used public OCT dataset^16^, as well as on an internal dataset, we demonstrate that this strategy allows us to achieve state-of-the-art accuracy, and we present ablation studies that illustrate the relative contribution of our individual architectural choices. Subsequently, we demonstrate that quantification of drusen based on these results exceeds the accuracy of drusen volume and localization compared to a previous state-of-the-art method for automated drusen segmentation.

## Material and methods

Previous work has compared different approaches to CNN-based drusen segmentation and found that first segmenting the retinal pigment epithelium (RPE) and Bruch's membrane (BM), and detecting drusen as abnormal deviations between them, gave more accurate results than trying to predict drusen with pixel-wise classification^4^. Our current work further strengthens this strategy by introducing a novel neural network architecture that allows us to replace a shortest path finding post-process, which was previously required to reconstruct one-pixel thick layers from two-dimensional segmentation maps, with direct end-to-end learning that permits localization with sub-pixel accuracy. This also accounts for an additional anatomical constraint, in particular, the correct order of the layers, and it allows for a further reduction of segmentation ambiguities by jointly learning the position of the ellipsoid zone (EZ). Finally, we refine the previously proposed method for estimating drusen from layer positions.

### Layer head

**Figure 1:**
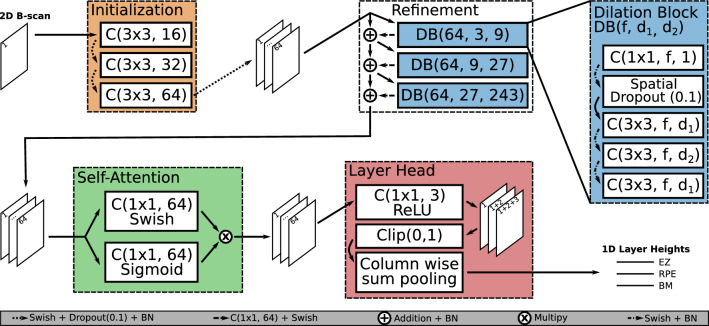
Our proposed model architecture. The Initialization block receives the input data and creates a feature space. This is handed over to the Refinement block which is built from multiple dilation blocks, to integrate global context into the feature space. The Self-Attention block scales features in our feature space with access to the global context and returns 64 output channels. The Layer Head transforms 2D feature maps into a 1D representation where in our case every 1D output channel represents the height of an OCT layer. Convolutions are indicated as C(kernel-size, filters) and dilation blocks as DB(filters, dilation-1, dilation-2).

We designed our model so that it directly predicts the layer heights and enforces the correct layer order, without requiring post-processing like shortest-path finding. Both goals are achieved with our Layer Head which can be attached to arbitrary semantic segmentation models and turns *N* 2D output maps into *N* 1D height maps which in our case refer to *N* OCT layer height maps (Fig. 1). It guarantees that predicted layers are without holes for the complete B-scan without any post-processing. The used summed squared error loss explicitly leads the model towards minimizing the distance between ground truth and predicted layers.

The main idea of the Layer Head is that, when all values above the layer position in a given image column (A-Scan) are 1 and all values below it are 0, the layer height, measured as the distance to the top of the B-Scan, can be computed as the column-wise sum. Moreover, we can guarantee the correct order of multiple layers by representing each of them in a separate channel, with earlier channels corresponding to higher layers, and taking cumulative sums along the channel axis. This way, constraining all values to be non-negative ensures that later layers are never estimated to be above an earlier one, and clipping pixel-wise sums greater than 1 ensures that layer positions remain bounded by the image height.

When using our Layer Head one has to keep in mind that good predictions depend on the model’s ability to determine for every pixel, whether it is below or above the searched height. This might require the receptive field of the model to cover the whole input image.

### Base model architecture

For the Layer Head to work, every pixel in the output map needs to be clearly identified as above or below the retina. This requires global image context. Common ways of increasing the receptive field size are pooling operations^17^ and strided convolutions^18^. However, they require suitable up-sampling operations to achieve segmentation at the input resolution.

For this reason, we build on work by Soullard et al.^19^, whose architecture we will refer to as MultiScaleGatedDenseNet, to implement a novel, fully convolutional model architecture that builds solely on dilated convolutions to increase the receptive field size. Our final model has 393.923 parameters in contrast to ~ 31 million parameters for the standard U-Net architecture^17^ and ~ 37 million parameters for the DeepLabV3 + architecture^18^, where the reduced resolution is compensated for by using large numbers of channels which greatly increases the number of parameters. Parameter-wise our model also compares favorably to especially small versions of the U-Net, such as the Squeeze U-Net^20^ with ~ 2.59 million parameters.

We adopted the use of multiple dilated convolution blocks with spatial dropout and the gating mechanism from the MultiScaleGatedDenseNet, but made several changes to the overall architecture as well as the individual dilated convolution blocks to obtain a model with the desired receptive field size and improved performance. Since the effective receptive field size is smaller than the calculated receptive field size^21^ we decided to overshoot the input image size to guarantee global context for every pixel in the output map. Our final architecture has a receptive field size of 721 to make predictions for an input shape of (512 × 512) pixels.

The rationale behind our base architecture is to create a robust feature space for every pixel which enables its classification. The use of dilated convolutions, which results in a constant size for all intermediate representations, allows us to iteratively refine this feature space by learning residuals. Our base architecture is explained in the next sections and the individual blocks are highlighted in Fig. 1. We use the Swish activation function instead of ReLU when not stated otherwise. This choice is based on an experimental comparison of different activation functions that showed that the Swish activation function “tends to work better than ReLU on deeper models across a number of challenging datasets”^22^.

#### Initialization block

The Initialization block is the first block of our model. Here we stepwise increase the number of channels using (3 × 3) convolutions from 1 input channel over 16 and 32, to 64 which is the dimensionality of the feature space we want to build. Every convolution is followed by a Swish activation function, a dropout layer, and batch normalization. We use dropout with a rate of 0.1 during training, which aims at making the initialization block more robust against noisy input data where features might be locally obscured.

#### Refinement block

In the second block, we refine the created feature space in a residual learning fashion in the sense of residual networks^23^, using dilated convolution blocks (DBs). Our DBs start with a 1 × 1 convolution and another 1 × 1 convolution is performed before the learned residuals are added to the feature space. This lets each DB choose for which maps in the feature space residuals are computed and what to base these residuals on. The first 1 × 1 convolution is followed by a spatial dropout layer with a rate of 0.1^19^. The spatial dropout switches off complete feature maps instead of individual pixels and aims at making the model robust against missing features on an image level. To increase the receptive field, dilated convolutions are used in an expanding and contracting pattern to integrate features from larger scales locally. For example, the first DB uses convolutions with dilation factors 3, 9, and 3. The DB is shown on the right in Fig. 1. The learned residuals are added to the input feature space and the sum is batch normalized before being fed to the next DB. This differs significantly from the MultiScaleGatedDenseNet, where the output of a block is concatenated to its input and then fed to the next block. After this refinement, every pixel had the opportunity to integrate information from different scales provided by the different dilated convolution blocks.

#### Self-attention block

The third part of our architecture uses the self-attention/gating mechanism from the MultiScaleGatedDenseNet on the refined feature space. It aims to focus the network on the information that is most relevant for its final task and works by element-wise multiplication of a Sigmoid and a Swish-activated linear combination of the refined feature space. Here the model can learn which features to suppress or emphasize for every pixel individually based on the global information collected in the refinement step. We apply this self-attention mechanism once, to produce the final features. At this point, the per-pixel features capture context from the full image via the refinement blocks. This gives the self-attention mechanism the opportunity to use global context.

### Training procedure

All our models are trained for 35 epochs with an exponentially decreasing learning rate which starts at 0.001 and is multiplied by exp(− 0.1) every epoch. We use the Adam optimizer with clipnorm = 1 and clipvalue = 0.5. The batch size is 2 and the data is shuffled in each iteration. The data is augmented on the fly by random horizontal flips. Additionally, the vertical position of the retina in the image is altered to be uniformly distributed in the training data. Therefore we first center the B-Scan to 75 pixels above the average BM height and then apply a random vertical shift between − 180 and 180 pixels. As a loss function, we use the summed squared error (SSE), which puts less weight on partially annotated B-scans than the mean squared error would.

### Drusen computation

Drusen are an important biomarker for AMD progression and can be detected based on an increased distance between retinal pigment epithelium (RPE) and Bruch's membrane (BM). Prior work has segmented drusen by fitting a 3rd-degree polynomial to the RPE annotation to estimate a healthy RPE. Those parts of the area between the fitted healthy RPE and the annotated RPE that passed a size-based false-positive elimination were marked as drusen^24^. Follow-up work improved this by an initial rectification of the retina using the BM^4^. Our experiments showed that fitting the 3rd-degree polynomial is not necessary after the rectification and might even be detrimental. Instead, we assumed that the healthy RPE has a fixed distance to the BM which might vary based on individual physiology and image resolution. To estimate this fixed offset we make a histogram of the rectified RPE height and compute the healthy RPE height as the average over A-scans within the mode of that histogram and its directly neighboring bins. To remove small false positives we filter drusen by their height, which is computed based on the complete volume. Therefore we determine the maximum height in connected components in an enface drusen projection. Drusen are removed if they are less than 2 pixels high.

### Data

**Figure 2:**
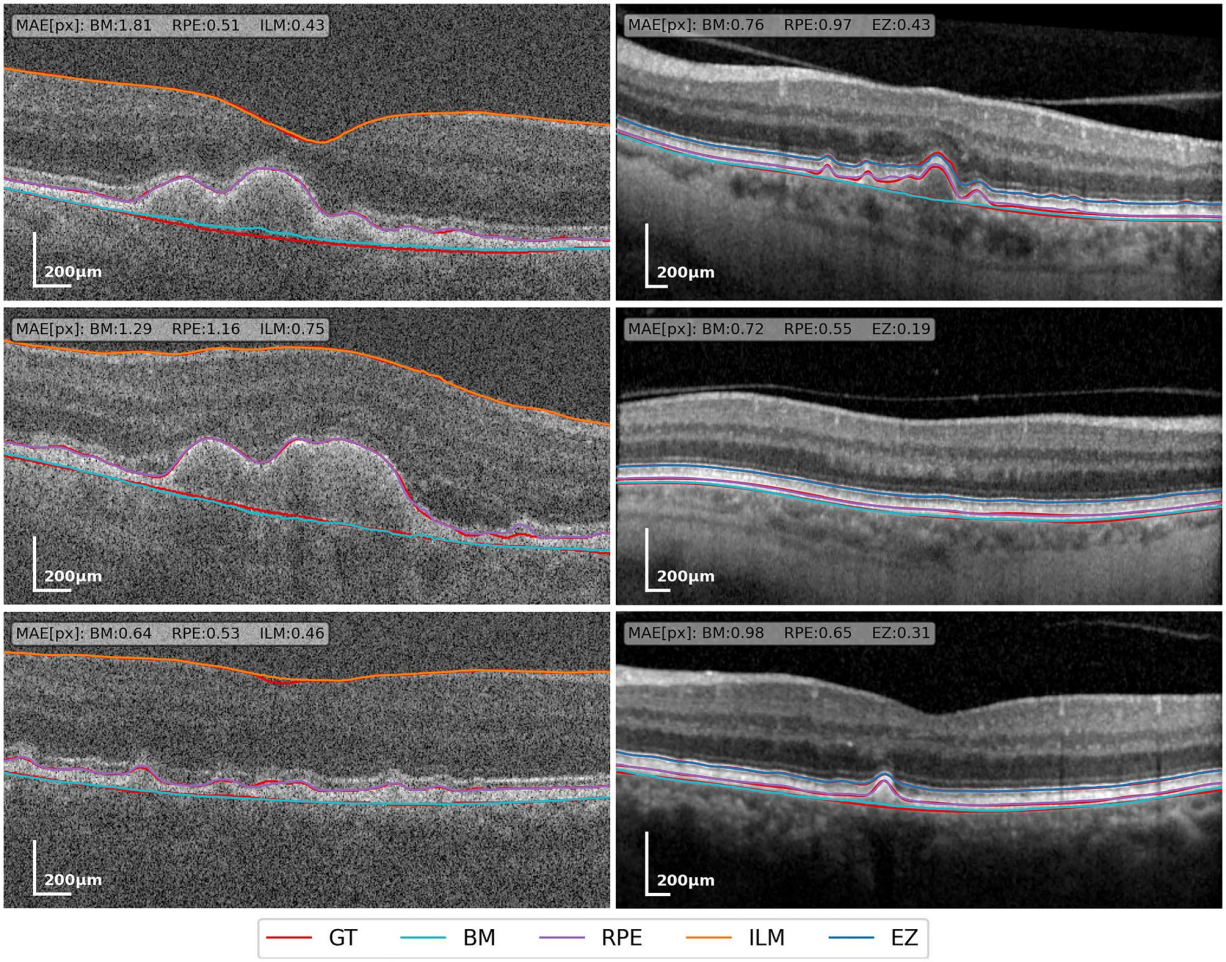
Exemplary B-scans from our validation data, Duke (Bioptigen) data on the left, internal data (Spectralis) on the right. Ground truth annotations are red. The remaining colors indicate layer positions estimated by our model. In the top left of each B-scan, the mean absolute error (MAE) in pixels is given for every layer.

Prior work indicates that the accuracy of algorithms for Drusen segmentation can depend significantly on the device with which images have been taken^25^. Therefore, we report results on two datasets, which were acquired with different devices. A qualitative comparison between the two datasets with ground truth and predicted annotations is shown in Fig. 2, illustrating differences in image quality and annotated layers. We used the eyepy Python package for processing and visualization of OCT data^26^.

The first dataset is an internal AMD dataset with expert annotations for BM, RPE centerline, and EZ^27,28^. All volumes were produced with a Spectralis device from HEYEX (Heidelberg Engineering, Germany) and have a resolution of 512 × 496. The study was approved by the Institutional Review Board of the University Bonn (approval ID 013/16). Written informed consent was obtained from all participants following an explanation of all study procedures. The protocol followed the tenets of the Declaration of Helsinki.

We split the dataset into training, validation, and test sets with 100 (12,886), 13 (1789), and 21 (2902) volumes and B-scans respectively. To evaluate our drusen segmentation, for each volume in the test set, drusen in 5 B-scans spread with equal distance over the central third of the volume were manually annotated independently by two readers using the EyeLab annotation tool^29^.

To compare our layer segmentation results to a wider range of prior approaches, we used a public OCT dataset from Duke University, acquired with a Bioptigen system^16^. It has annotations for BM, the inner boundary of the RPE, and the ILM for 115 Control and 269 AMD volumes. All volumes have 100 B-scans of size 1000 × 512 but the annotations are only available for a 5 mm diameter centered at the fovea. We selected the central 512 A-scans from every B-scan with more than 256 annotated A-scans per layer. B-scans with incorrect segmentations were removed from the training and validation data when they came to our attention. We did not remove any problematic B-scans from our test data, to have a fair comparison to previous work. This procedure resulted in 164 (11,427), 20 (1389), and 200 (14,228) volumes and B-scans in the training, validation, and test set respectively. These split ratios are the same as in prior work^5,13^ a list of excluded B-scans, the exact data split, and our trained models, are included with our published code.

No studies on humans or animals were performed by the authors for this article. Existing datasets were used for which all required ethics votes as well as informed participant consent had been obtained^16,27,28^.

All data is normalized to zero mean and unit variance before being fed into the model.

## Results

### Layer segmentation

On our internal dataset we compared a model predicting 2 layers (BM and RPE) against one predicting additionally the EZ. As a metric, we use the mean absolute error (MAE) between ground truth and prediction, rounded to integers. Reported mean and standard deviations are computed over the mean MAE per volume. The model predicting 3 layers performs slightly better while for both models the BM prediction (0.63 and 0.66) is better than the RPE prediction (0.85 and 0.88) and the EZ prediction (0.44) is the best (Table 1).

**Table 1:** Mean absolute differences and standard deviation in pixel over all A-Scans in the test dataset.

	AMD		
	EZ	RPE	BM
2 classes	–	0.88 ± 0.28	0.66 ± 0.20
2 classes BCI 95%	–	[0.67, 1.01]	[0.58, 0.74]
3 classes	0.44 ± 0.09	**0.85 ± 0.28**	**0.63 ± 0.19**
3 classes BCI 95%	[0.35, 0.56]	[0.74, 0.96]	[0.55, 0.70]

Results suggest that a 3-class setup, in which the network is trained to also segment the ellipsoid zone (EZ), slightly supports segmentation of the retinal pigment epithelium (RPE) layer and Bruch’s membrane (BM). The 95% BCI was calculated via bootstrapping, randomly resampling our test dataset 10,000 times, with replacement. Each sample is 80% of the size of the full test dataset.

Best values are in [bold].

Our final model outperforms three approaches that previously reported results on the public dataset from Duke University^16^ with regard to the MAE (Table 2). Segmentation results are slightly better in the control volumes than in the AMD volumes while in both parts the ILM is segmented best and the BM worst. Additionally to the standard deviation we report a bootstrapped confidence interval (BCI) of the mean.

**Table 2:** Mean absolute differences and standard deviation in pixel over all A-Scans in the test dataset.

	Control			AMD		
	ILM	IBRPE	BM	ILM	IBRPE	BM
Shah et al.^12^	1.04 ± 0.07	1.19 ± 0.18	1.54 ± 0.31	1.15 ± 0.25	1.88 ± 0.57	1.81 ± 0.56
Liefers et al.^13^	0.840	1.280	1.227	1.055	1.568	1.858
Asgari et al.^5^	0.65 ± 0.06	1.06 ± 0.12	0.9 ± 0.08	0.88 ± 0.09	1.23 ± 0.11	1.15 ± 0.1
Model A (no layer order)	0.39 ± 0.42	0.72 ± 0.39	0.85 ± 0.69	0.48 ± 0.75	0.77 ± 0.57	1.38 ± 3.02
Model B (no attention)	0.77 ± 1.45	0.76 ± 0.39	0.92 ± 1.24	0.91 ± 2.50	0.78 ± 0.59	1.22 ± 0.97
Model C (no shortcus)	0.33 ± 0.40	0.76 ± 0.48	0.86 ± 1.16	**0.37** ± 0.61	0.79 ± 0.72	1.15 ± 0.89
Final	**0.31** ± 0.31	**0.71** ± 0.41	**0.74** ± 0.47	**0.37** ± 0.67	**0.72** ± 0.66	**1.08** ± 0.70
Final BCI 95%	[0.26, 0.35]	[0.64, 0.79]	[0.67, 0.81]	[0.27, 0.47]	[0.61, 0.83]	[0.95, 1.20]

Results are shown for three previous works, our final model, and 4 ablation studies where our model was trained without guaranteed order (A), without the self-attention block (B), and without shortcut connections (C). The 95% BCI was calculated from 10,000 resamples as above.

Best values are in [bold].

### Ablation studies

To show the effectiveness of our architectural choices, we performed an ablation study in which we trained 3 variants of our model on the Duke dataset. All models were trained with the same loss, and the exact same hyperparameters as the final model, except for the ablated characteristics. Model A removes the cumulative channel sum in the Layer head that guarantees the layer order. Model B has no self-attention block, hence the output of the refinement block is fed directly to the Layer Head. Model C removes the shortcut connections that implement a residual learning strategy in the refinement block. Our final model worked better than all three alternatives from the ablation study.

### Drusen segmentation

We evaluated our proposed drusen segmentation that builds on the three-layer model with respect to two experts and compared results to a previous method for layer-based drusen segmentation^4^. Our metric for the segmentation quality is the Dice score (DSC), a commonly used similarity measure that relates the size of an intersection of two sets to their combined size.

$$DSC = \frac{2 |X \cap Y|}{|X| + |Y|}$$

where $X$ and $Y$ are segmentations.

**Figure 3:**
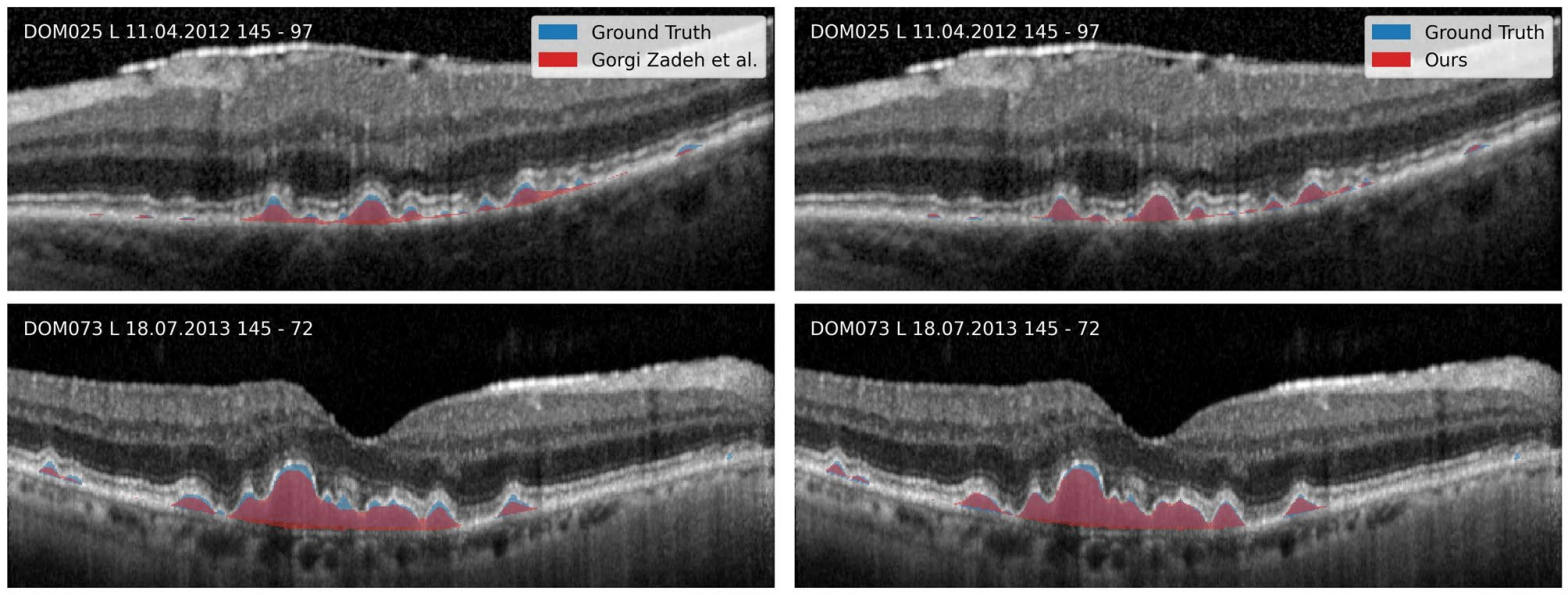
Comparison between automatic and manual drusen segmentation. Previous work is shown on the left and our results are shown on the right. Our results clearly reduce issues of under segmentation in the previous method.

The inter-reader agreement in terms of dice score was 0.67 ± 0.19. With respect to Reader 1, who also annotated the layers in our training dataset, our proposed method achieves a drusen Dice score of 0.71 ± 0.16, which improves upon the 0.60 ± 0.23 for the previous method by Gorgi Zadeh et al.. Dice scores with respect to Reader 2 are lower, but with 0.62 ± 0.23 instead of 0.53 ± 0.25, our method still improves over the previous one. Qualitative results are shown in Fig. 3.

### Drusen volumes

For any downstream analysis, the drusen load obtained by automatic approaches is required to have a high correlation with manually obtained results. To verify this, we computed the Pearson correlation between drusen volumes obtained manually and automatically for OCT volumes in our test data (Table 3). We found that the correlation is high overall, with our model correlating better with both human readers than the prior U-Net based approach^4^, while the inter-reader correlation is falling behind in comparison.

**Table 3:** Pearson correlations between drusen volumes in our test set. Drusen volumes based on our model have a higher correlation with manual gradings for both readers than those based on a previous U-Net based method^4^.

	Reader 2	U-Net	Ours
Reader 1	0.984	0.989	**0.994**
Reader 2		0.983	**0.988**
U-Net			0.998

Best values are in [bold].

**Figure 4:**
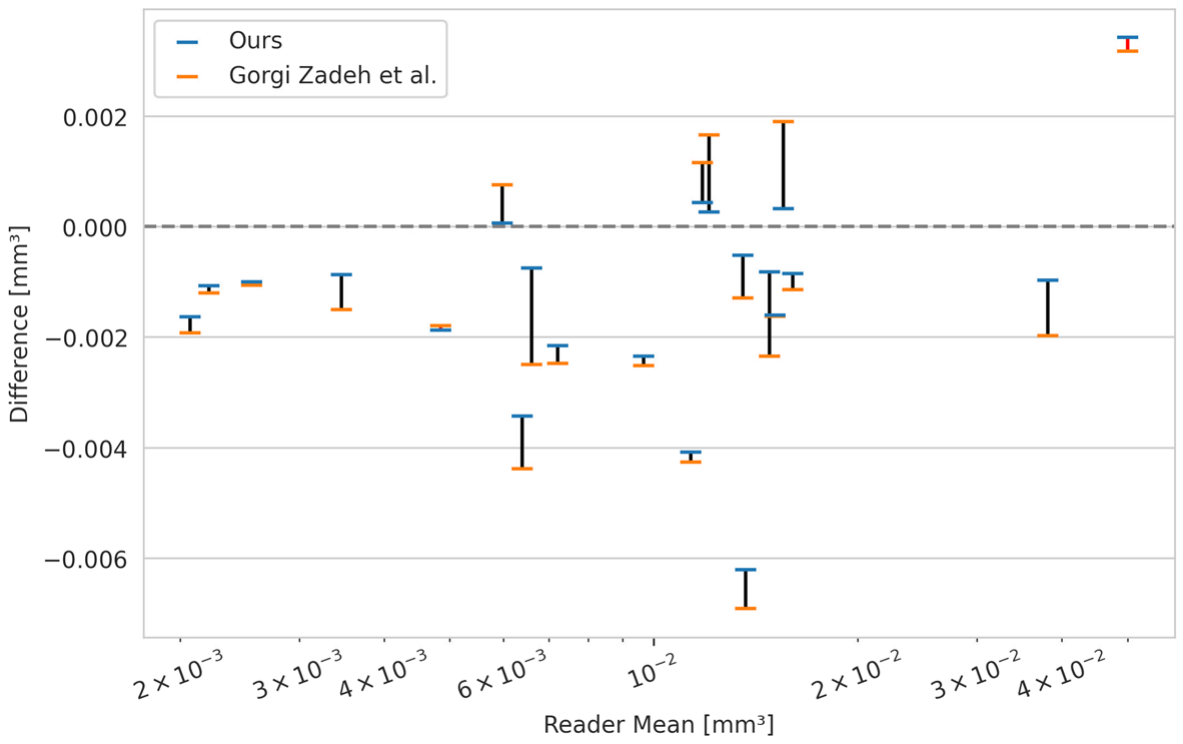
Drusen volume differences for two automated methods. Differences are computed between two automatic approaches and the mean drusen volume from two human readers for every volume in our test data. Datapoints for the same volume are connected with a black line if our method's volume difference is closer to 0 and with a red line otherwise. In 19 out of 21 volumes the results of our method are closer to the reference.

Additionally, absolute volume differences between human readers and automatic drusen quantification should be small. To assess this, we computed the difference between results from both methods and a reference that is created by averaging the volumes of both human readers (Fig. 4). While both methods tend to underestimate the drusen volume, our method is closer to the reference in 19 out of 21 volumes.

## Discussion

In this work, we introduced a novel algorithm for automated drusen segmentation in OCT images. We showed that our method outperforms a previous method in terms of both the correlation with manual grading and the difference to the mean volume of two human readers. We also showed that our method reproduces the results on an independent test set that was annotated by the same reader as the training data more accurately than a second reader. We conclude that our method’s accuracy is sufficient for use in epidemiological studies, and believe that this result further illustrates the importance of objective assessment. We demonstrated improved accuracy compared to a previous method by Gorgi Zadeh et al., which already quantified drusen reliably enough to find associations with visual function measures^28^. Making a prediction for a B-scan of size 496 × 512 took, on average, ~ 105 ms on an Nvidia Titan X (Pascal) GPU. We believe that our method will be useful for future epidemiological studies and clinical trials that aim to quantify drusen load and thereby provides a basis for insights into their role in AMD.

The most important factor contributing to the improved performance of our method is the improved layer segmentation. In particular, the results indicate a more accurate RPE in the AMD group, which is crucial for drusen segmentation. Among technical refinements such as an attention mechanism and residual learning, our ablation study shows the importance of exploiting additional anatomical knowledge by enforcing the layer order. This results in a model that does not require post-processing via shortest path approaches for generating layer heights and has fewer parameters than previous models while yielding more accurate results.

A limitation of our current approach is that it does not clearly indicate regions in which layers are not present. Anatomical regions without a meaningful layer structure such as the optic disc can be filtered by restricting the domain of subsequent analysis. Filtering regions without a layer structure due to severe atrophies would however require an extension of our method, and training on a sufficient number of examples.

## Conclusion

We presented a specialized model for OCT layer segmentation that predicts layer heights in an end-to-end fashion and allows for accurate layer-based drusen segmentation. The drusen volume based on our prediction has a high correlation with manual gradings and improves upon an established previous method. We are confident that our model will improve the assessment of AMD-related retinal changes on OCT in large-scale epidemiological studies as well as help clinicians with the assessment of individual patient data.

## Data Availability

Model weights and a command line tool for using the model will be accessible at time of publication in our Github repository www.github.com/MedVisBonn/eyeseg. The Duke data was obtained from the official homepage: https://people.duke.edu/~sf59/RPEDC_Ophth_2013_dataset.htm.
